# Employing Supervised Algorithms for the Prediction of Nanomaterial’s Antioxidant Efficiency

**DOI:** 10.3390/ijms24032792

**Published:** 2023-02-01

**Authors:** Mahsa Mirzaei, Irini Furxhi, Finbarr Murphy, Martin Mullins

**Affiliations:** 1 Department of Accounting and Finance, Kemmy Business School, University of Limerick, V94PH93 Limerick, Ireland; 2 Transgero Limited, Newcastle West, V42V384 Limerick, Ireland

**Keywords:** nanomaterials, nanoparticles, machine learning, antioxidant, reactive oxygen species

## Abstract

Reactive oxygen species (ROS) are compounds that readily transform into free radicals. Excessive exposure to ROS depletes antioxidant enzymes that protect cells, leading to oxidative stress and cellular damage. Nanomaterials (NMs) exhibit free radical scavenging efficiency representing a potential solution for oxidative stress-induced disorders. This study aims to demonstrate the application of machine learning (ML) algorithms for predicting the antioxidant efficiency of NMs. We manually compiled a comprehensive dataset based on a literature review of 62 in vitro studies. We extracted NMs’ physico-chemical (P-chem) properties, the NMs’ synthesis technique and various experimental conditions as input features to predict the antioxidant efficiency measured by a 2,2-diphenyl-1-picrylhydrazyl (DPPH) assay. Following data pre-processing, various regression models were trained and validated. The random forest model showed the highest predictive performance reaching an R^2^ = 0.83. The attribute importance analysis revealed that the NM’s type, core-size and dosage are the most important attributes influencing the prediction. Our findings corroborate with those of the prior research landscape regarding the importance of P-chem characteristics. This study expands the application of ML in the nano-domain beyond safety-related outcomes by capturing the functional performance. Accordingly, this study has two objectives: (1) to develop a model to forecast the antioxidant efficiency of NMs to complement conventional in vitro assays and (2) to underline the lack of a comprehensive database and the scarcity of relevant data and/or data management practices in the nanotechnology field, especially with regards to functionality assessments.

## 1. Introduction

Reactive Oxygen Species (ROS) are molecules that contain one or more unpaired electrons in their orbitals and can lead to the formation of free radicals, e.g., superoxide (O_2_•−), hydroxyl (•OH), peroxyl (ROO•) or hydrogen peroxide H_2_O_2_. ROS are generated during normal cellular functions as a by-product of aerobic metabolism and are essential to regulate physiological functions in biological systems [1]. However, exposure to exogenous factors such as heavy metals or ionizing radiation can trigger the excessive generation of ROS. The disruption of the equilibrium of ROS leads to oxidative modifications of biological systems at a molecular level, causing damage to cellular components, such as DNA, lipids and proteins. Moreover, it leads to the acceleration of cellular death which underlies several diseases [2]. Therefore, it is necessary to reduce or eliminate the excess of free radicals. Free radicals can be eliminated or detoxified by a selection of antioxidative enzymes or antioxidants [3]. Therapeutic approaches for preventing oxidative damage are currently at the core of medical research, including the use of antioxidants [4].

The antioxidant efficiency (anti-ox) is defined as the ability of molecules to scavenge the free radicals. Antioxidant compounds (natural or synthetic) control autoxidation by preventing the formation of free radicals or interrupting their spread by neutralizing them which delays or inhibits the damage to the target cells, decreasing oxidative stress, enhancing immune function and increasing longevity [5,6]. Numerous studies have investigated the anti-ox properties of various materials [7,8,9]. Conventional antioxidants are designed to reduce ROS in biological systems [10]. However, clinical investigations have shown that traditional molecules provide little to no protection or even, in some instances, increase the risk of mortality [11]. Ghorbani [12] demonstrated that antioxidants show inadequate effects due to their unspecific distribution and poor retention in disease sites. Therefore, the need for an efficient, safe yet more specific antioxidant is undeniable.

Nanotechnology has gained tremendous attention due to its potential to treat diseases and it is growing rapidly within the medicinal disciplines [13,14,15]. Nanomaterials (NMs) are particles with unique features such as a high surface-to-volume ratio, biocompatibility, redox and catalytic properties and are used in many fields such as the pharmaceutical industry, textile, cosmetics, food, water treatment, and particularly in health-related biological fields [16,17,18,19]. Nanomedicines overcome limitations such as non-specific distribution, high renal clearance, low delivery efficiency and inadequate in vivo bioavailability [20,21].

There are numerous organic and inorganic NMs exhibiting strong ROS-scavenging activities [22,23]. Studies have demonstrated promising results in developing a novel class of antioxidants (nano-antioxidants) to overcome the challenges associated with conventional antioxidants [12]. The potential use of NMs to reduce damages related to oxidative stress and prevent diseases is the subject of intense scientific research [20,24]. The potential use of NMs in treatments of degenerative Alzheimer’s disease, cancer, metabolic diseases and chronic inflammatory diseases has been reported [25,26]. Ghorbani [12] demonstrated that NMs have stable anti-ox activity with specific pharmacokinetic actions, intrinsic ROS-scavenging properties and fewer adverse effects.

In recent decades, there has been considerable interest in digitising the information in medical and pharmaceutical disciplines and the use of such data to explain biological phenomena [27]. As more data becomes available, the application of artificial intelligence (AI) becomes increasingly valuable for analysing the data. AI technology, implemented in various fields, e.g., molecular medicine, drug development and nanotechnology, provides great reproducibility and timeliness to accomplish a variety of tasks [28,29,30]. This approach is data intensive [31] and has been applied in fields of medicine including disease prediction [32], the discovery of new medicine [33], the prediction of drug responses [34], medicine’s toxicity [35], nanotoxicity [36], antimicrobial resistance [37], antibiotic resistance [38] and the antibacterial capacity of NMs [39], to mention a few. In the era of data, machine learning (ML) models, a sub-field of AI, can improve the predictions of toxicological and functional effects and provide insights into the design of NMs and/or strategies to eliminate exposure and minimize risks, contributing to sustainable and safe-by-design approaches (SSbD) [31,40,41]. The ML approach is a promising tool as it is time- and cost-effective reducing the number of tests required while allowing the researcher to be more effective by providing them precise forecasts. Furthermore, the traditional risk assessment is transforming into digital frameworks consisting of new approach methodologies (NAMs) without the need for animal testing [42,43].

ML models have been used to predict anti-ox activities, for example, Karydas [44] successfully developed an ML model to assess cherries’ free radical scavenging capacity where in their analysis they utilised the 2,2-diphenyl-1-picrylhydrazyl (DPPH) and folin-ciocalteu (FCR) lowering capacity as outputs while sensing indices, soil attributes, climatic, topographic and hydrographic data were used as input features. They employed and achieved an extreme gradient boosting (XGBoost) algorithm with an accuracy of R^2^ = 0.89. They employed important attribute analysis using the F-score method and determined the variables that affect the antioxidant capacity of cherries. Another example is from Idowu and Fatokun [45], which examined supervised and unsupervised algorithms to correlate polyphenol’s antioxidant activity (output feature) to molecular descriptors, with parameters regulating in vivo antioxidant behaviour (input features). Olsen [46] demonstrated the use of a deep convolutional neural network (CNN) to predict the anti-ox efficiency measured by a DPPH assay based on the properties of 687 peptides. They demonstrated that the model can be used to estimate the potential of a protein’s peptides to scavenge free radicals.

Given the time-consuming, expensive and work-intensive nature of NMs’ synthesis, having a computational method that can predict the anti-ox properties is beneficial. In addition, due to the large number of NMs’ P-chem possible combinations and the impact of experimental variables, it is not feasible to evaluate all of them using conventional laboratory tests to determine the ones with the highest capacities. In this work, we manually compiled a dataset derived from a literature review of in vitro studies in different mediums that can be used by other researchers to train models, augment the dataset with additional features, fill data gaps through grouping approaches and predict the efficiency of NMs to scavenge free radicals. Despite the limited data available [47], we explored ML tools and we were able to predict the anti-ox efficiency of NMs and we also demonstrated which attributes have a substantial impact on the predictions. To the best of our knowledge, no study has been undertaken on the use of ML tools to predict the scavenging efficiency of NMs. This novel study contributes significantly to the existing research by proposing a model that can fit the SSbD framework, in the functionality dimension [31]. This tool utilizes P-chem properties, exposure conditions and the method of NMs’ synthesis preparations. A random forest algorithm demonstrated the highest accuracy, reaching an R^2^ = 0.83.

## 2. Results

### 2.1. Data Pre-Processing

Due to high missing values among the input-related data collected, we selected NMs’ type, core size, shape, dosage, coating as well as the synthesis process, medium used, absorbance and duration as the input features for the model. This data cleansing resulted in a final dataset consisting of 10 columns and 1027 rows. Table 1 shows the percentages of missing values among the initial input variables: core size (3%), HDS (78.30%), shape (15.68%), EE (90.34%), ZP (65.09%), PDI (82.05%), SA (99.51), dosage (9.86%), temperature (76.33%), pH (98.62%) and thermal stability (86.59%).

The HDI, EE, PDI, SA, ZP, temperature, pH and thermal stability columns were excluded due to the presence of numerous missing values. Null data in core size, dosage and shape were replaced with the mean (for numeric features) or mode value (for nominal features) [48]. Additionally, to avoid overfitting the model, the coating was transformed to a binary feature (coated and uncoated).

### 2.2. Model Validation

**Figure 1:**
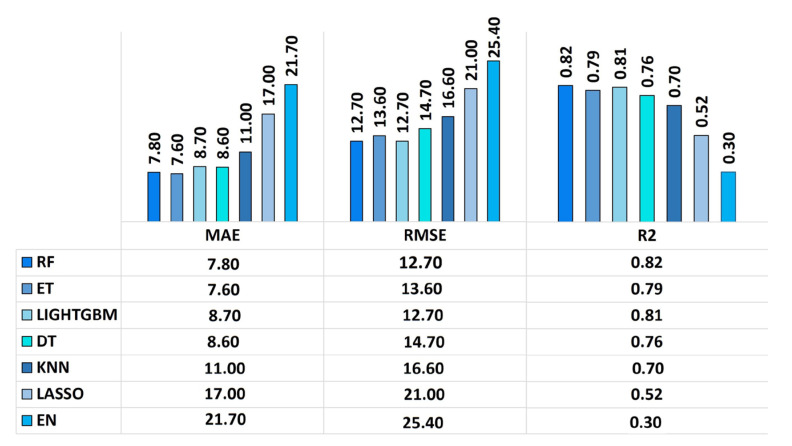
External models’ validation metrics from the test dataset (30%). Comparison of the seven regression models (columns used for this evaluation includes type of NMs, medium used, core-size, shape, synthesis techniques, coating, dosage, absorbance, duration.

Following data homogenization and pre-processing, we trained and compared several regression models. The models are trained with the training dataset comprising 70% of the data (719 rows). Internal 10-fold cross validation is performed to optimize the hyper parameters and to ensure the models’ robustness and goodness-of-fit. Following the model training, we performed model validation using 308 rows out of 1027 (30% of data) as a test set (external validation) to evaluate the final tuned predictivity. As shown Figure 1, the external validation of the RF model outperformed the other regression models exhibiting the lowest error with MAE at 7.8, RMSE at 12.7 and the highest accuracy displaying a R^2^ = 0.83.

Nonlinear regression models (RF and ET) outperform linear models (LASSO and EN) because they exhibit more parameters (properties of training data learned during the learning process). RF is excellent at processing categorical, binary and numeric features, making it ideal for working with high-dimensional data with minimal pre-processing requirements.

**Figure 2:**
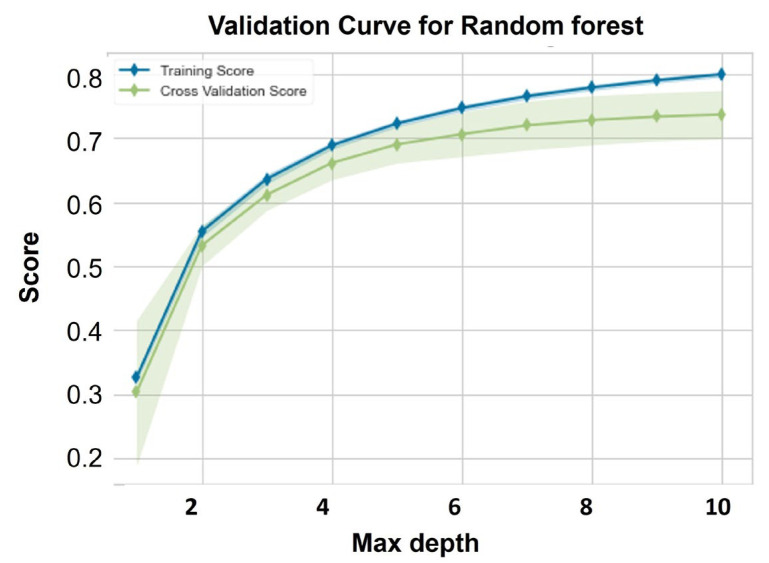
The internal model validation curve of RF model for the training score and the cross-validation score. x-axis represents the hyperparameter of the tress depth, y-axis represents the score (accuracy) of the model.

Figure 2 presents the validation curve of the RF algorithm derived from the internal validation dataset (70%), the values of the training and cross-validation score are close, indicating the model goodness of fit and robustness of the training model.

**Figure 3:**
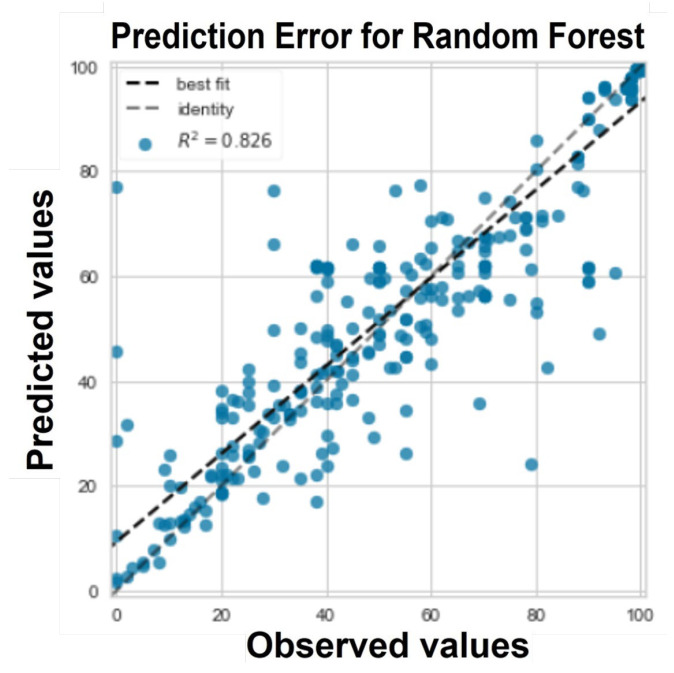
The prediction error by the RF regression model, the x-axis represents the actual (observed) values and the y-axis represents the predicted values.

Depicted in Figure 3are the prediction error of the predicted values against the actual values. It shows that the predicted values produced by our model (0.83) are close to the observed targets from the dataset. In regression models, prediction error measures how effectively the model predicts the response variable and as can be seen, the predicted values are relatively close to the actual values.

### 2.3. Model Prediction

#### Attribute Importance

**Figure 4:**
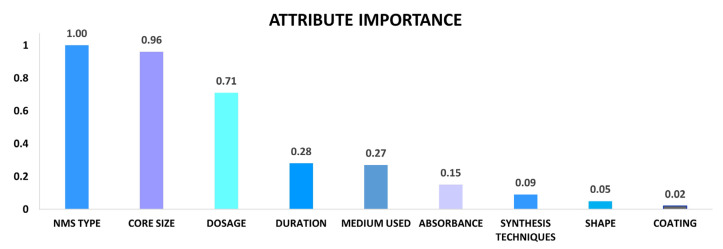
Attribute importance generated by random forest model, y-axis represents the variable importance from 0 to 1, x-axis represents the attributes.

The feature importance in the RF model is calculated using the impurity Gini-based feature importance (or Mean Decrease in Impurity). Figure 4 presents the attribute importance analysis performed by RF. As illustrated, NMs’ type, core-size and dosage appeared as the top three most important attributes influencing the prediction of the outcome.

The duration of exposure, the medium used and the absorbance are relatively important attributes that affect the prediction. The synthesis techniques, shape and coating (presence or absence) have relatively less effect on the prediction.

## 3. Discussion

The purpose of this research was initially to (i) gather data from the literature related to the anti-ox efficiency of NMs, (ii) to present the landscape of data gaps related to functionality assessments, (iii) to apply a tool to demonstrate the feasibility of ML for this outcome and iv) to identify significant factors influencing the prediction of the outcome.

Tremendous efforts are being put together to create databases with complete datasets and also many initiatives to promote the FAIR (findable, accessible, interoperable and reusable) principles are being created in the nano-domain [49,50]. To the best of our knowledge, there is no publicly accessible database that collects and summarizes data on the P-chem characteristics, functionality, application and production of NMs and at the same time links this information to safety aspects. Such a database could enhance the correlation between functionality and safety and thus support the SSbD paradigms [51]. In this study, we manually built a dataset that adheres to the FAIR principles: it is findable (10.5281/zenodo.6584826, accessed on 26 May 2022), accessible (open access), interoperable (containing the methodology and source of data as well as experimental parameters) and reusable in order to aid data scientists to accelerate the nano-informatics field. We collected data on P-chem experimental settings, such as TEM, SEM, FESEM, HRTEM, XRD, EDX, UV–vis spectroscopy and FTIR, and employed them to measure and evaluate intrinsic properties such as size, morphology, chemical composition and extrinsic properties such as encapsulation efficiency, polydispersity index and zeta potential. The various methodologies have an impact on the features’ quality and homogeneity of information. However, due to the lack of data, we utilized all the available information. When more data becomes available and FAIR, attributes generated through one technique will reflect more scientifically sound choices for these modeling purposes.

To evaluate the free radical scavenging capacity, we gathered data from six different methods such as DPPH, ABTS, NO, H_2_O_2_, OD and metal chelating. However, we selected the DPPH assay as the outcome due to substantial missing values among the other techniques. According to the literature, ABTS and DPPH assays are recommended with ABTS considered lengthy and expensive. It is challenging to conclude which approach is the most suitable for assessing the anti-ox activity. Bedlovičová [52] examined DPPH and ABTS assays, and they concluded that DPPH assay is inexpensive and simpler. When more information becomes available it could be feasible to utilize all the assays under one dataset if the parameters affecting the measurements become transparent with an increased variability due to data size. Another factor regarding the quality of data is the comparable measurements (unit metrics), which makes it difficult to merge a homogeneous dataset that contains all the knowledge gained through valuable experimental research. This may obstruct the advancement of a data-driven research. Thus, our work points out the shortcoming in current data management practices and reporting schemes in the field of nanotechnology. A uniform and homogenous system for reporting data on properties and experimental conditions will help to improve the quality of data and completeness.

Regarding P-chem characteristics, only 35%, 22%, 10%, 18% and 1% of the selected studies reported ZP, HDS, EE, PDI and SA of NMs, respectively. Therefore, due to a substantial number of missing values, we were unable to incorporate all these data. Therefore, for this study we employed the type of NM, medium used, core-size, shape, synthesis techniques, coating, dosage, absorbance, duration and DPPH variables. However, the performance of the models was compared by including ZP values computed with an ML missing values imputation methodology, and/or including shape (Supplementary Materials, Tables S1–S3). These properties are important in determining the activity of NMs. Regarding the core size, dosage and shape, the missing values were replaced with the mean or mode value. Thus, we stress that future research should report as many P-chem properties as feasible to allow the usage of intrinsic and/or extrinsic properties information. For experimental settings, we obtained data on the medium used, temperature, pH, thermal stability, spectrometers absorbance and exposure duration. However, the majority of them were excluded due to substantial number of missing values.

We utilized a model that adheres to OECD (organization for economic co-operation and development) principles (https://github.com/mahsa-mirzaei/NMAOP_M-M_wo-ZP-/blob/main/AOP_M%26M_wo%20ZP%20.ipynb, accessed on 18 January 2023), has a well-defined approach and is validated and built to an explicit endpoint derived from one assay [53]. The RF model surpasses all other models, with the highest R^2^ value, R^2^ = 0.83. In the nano domain, ML tools have been implemented to predict and assess the toxicity and functionality of NMs [54]. Furxhi [55] investigated NF’s toxicity, whereas Mirzaei [39] and Mirzaei [56] assessed the antibacterial properties of NMs as such or coated on textiles, respectively. However, to our knowledge, no similar studies that predict the anti-ox capacity of NMs have been published. This approach is the initial step toward assisting researchers in discovering NMs with a high capacity and determining their P-chem properties and/or experimental settings. Despite these limitations, our findings are congruent with the previous literature [57,58,59]. The RF model’s attribute importance analysis showed P-chem characteristics including type and core-size as influential variables on the prediction of the outcome, followed by shape, zeta potential and synthesis techniques. Researchers have investigated the anti-ox efficiency of numerous NMs and discovered that properties including particle core size, chemical composition, surface charge and surface coating have an impact on their anti-ox capacity [60,61,62,63]. According to Sharpe [57], the type of NM, core-size and dosage are among the most pivotal components. Fafal [59] stated that different synthesis techniques can affect the anti-ox capacity of NMs, they demonstrated that green techniques could have relatively higher activity. Nevertheless, the other characteristics such as ZP, EE and PDI can be equally valuable and play an important role. A study by Ruktanonchai [64], indicated that the ZP is a useful tool to predict the physical stability of NMs, and the PDI can show the size distributions. Kim [58] identified that particle-size, ZP, EE and PDI can enhance the release property and as a result improve the activity. One limitation of our study is the categorical representation of NMs in our dataset which are converted into binary representation through one-hot encoding. The categorical attributes can be changed only when the chemical composition of the NMs is available or swapped with theoretical descriptors. Future studies should focus on the accurate representation of the intrinsic properties related to the composition, in order to be used with ML applications.

Significant numbers of NMs have been evaluated to date, resulting in a large pool of information scattered through the literature; it becomes increasingly challenging to analyze all NMs using traditional test methods. Accordingly, the demand for computational methods increases. We investigated inorganic and organic NMs to develop a model with good predictability (R^2^ = 0.83). In addition to complementing conventional in vitro testing, this approach is substantially more cost-effective and time-efficient. The capacities of algorithms can be exploited to fulfill new approach methodologies [65]. Once safety can be integrated with the functionality of NMs, the modeling tools will allow the development and optimization of NMs with desired properties while ensuring worker, public and environmental safety, and functionality.

## 4. Materials and Methods

**Figure 5:**
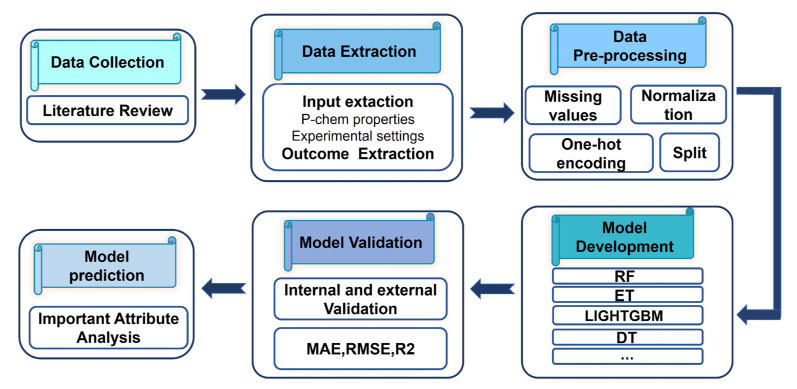
Model development workflow.

Figure 5 depicts an overview of the workflow followed. First, a literature review of studies investigating the efficiency of NMs via in vitro experiments was performed. P-chem properties of NMs, exposure conditions, measurement equipment-related features, characterization methodologies and assays used for assessing the anti-ox efficiency were extracted. Following an investigation of the dataset’s completeness, data pre-processing was carried out, including normalization [66], one-hot encoding [67] and data split [68]. Several regression models were trained and validated using a variety of performance parameters in order to identify a model with high predictability. To train ML models, we used Scikit-learn and PyCaret library [69]. Finally, importance attribute analysis, a supervised technique that identifies and ranks the variables was used [70].

### 4.1. Data Collection

In order to compose a high-quality dataset, a great amount of data must be integrated in a harmonized fashion which is the most challenging aspect of the ML workflow. A literature search was conducted for studies that investigated NMs’ antioxidant efficiency with quantitative results from 2013 to 2021. Following keywords were used ‘antioxidants’, ‘free radicals’, ‘ROS’, ‘Oxidative stress’, ‘RNS’, ‘nanoparticles’, ‘nanoforms’ and ‘nanomaterials’. We selected 73 studies that investigated different organic and inorganic NMs with in vitro experiments. We excluded 11 studies due to binary or qualitative results. From the studies gathered, the following information was manually extracted:

Input-related collection: (1) information related to NMs’ P-chem properties such as the NM type, core-size, coating, hydrodynamic size (HDS) and its polydispersity index (PDI), shape, surface area (SA), zeta potential (ZP) and encapsulation efficiency (EE); (2) experimental conditions such as the methods of NMs’ synthesis such as chemical (top-down and bottom-up approach) and green (bottom down approach employing natural extracts or microorganisms and biological components as reducing agents), the culture medium for the anti-ox activity evaluation, information related to the application of the NF (pharmaceutical and food packaging, nanomedicine, etc.), pH, temperature, thermal stability, absorbance and duration of the test. In addition, the characterization methodologies used, i.e., scanning electron microscopy (SEM), field emission scanning electron microscopy (FESEM), transmission electron microscopy (TEM), high-resolution transmission electron microscopy (HRTEM), X-ray Diffraction (XRD), energy dispersive X-ray (EDX), Fourier transform infrared spectroscopy (FTIR), dynamic light scattering (DLS) and Ultraviolet–visible spectroscopy (UV–vis spectroscopy) to evaluate the P-chem properties were collected.

Output-related collection: various assays can be used to assess the anti-ox efficiency of NPs including 2,2-diphenyl-1-picrylhydrazyl (DPPH), 2,2′-azino-bis (3-ethylbenzothiazoline-6-sulfonic acid (ABTS), nitric oxide (NO) or total radical-trapping antioxidant potential (TRAP). After collecting the above information, we selected the DPPH assay that measures the anti-ox efficiency of NMs as the output to be predicted, since it is the most popular, rapid, inexpensive and simple test [69]. In addition, the DPPH assay appeared as the most frequent assay (resulting in sufficient data points for modelling purposes) in the literature search and by choosing one assay we reflect the homogeneity of the experimental data measurements.

### 4.2. Model Development

Data pre-processing: PyCaret executes missing value imputation and categorical one-hot encoding. The missing values in the numerical features are replaced with the mean value of the features in the training dataset, whereas the missing values in categorical features are imputed with the most frequent or the mode value [70]. Rafsunjani [48] compared several imputation techniques and found that mean imputation performed the best for handling missing values. One-hot encoding was performed since regression models cannot handle categorical features. Normalization was used to rescale numerical values to reduce the impact of magnitude in the deviation without altering the differences in the range of values. The dataset was split into training (internal, 70% of the dataset to fit the model and to test goodness of fit) and test set (external, 30% of the dataset for evaluation of predictability).

Model development: We trained several regression models including Random Forest (RF), Light Gradient Boosting Machine (LIGHTGBM), Extra Trees (ET), Decision Tree (DT), K-Nearest Neighbors (KNN), Least Absolute Shrinkage And Selection Operator (LASSO), Elastic Net (EN) Least Angle Regression (LAR), passive aggressive regressor (PAR), Lasso Least Angle Regression (LLAR), Huber Regressor (HUBER), Adaboost Regressor (ADA), Bayesian Ridge (BR), Ridge Regression (RIDGE), Gradient Boosting Machine (GBM) and Orthogonal Matching Pursuit (OMP). All models were evaluated and compared to find the best model with good predictivity. The following seven models are selected for reporting in this study: RF is a supervised algorithm that employs both decision trees and bagging techniques. These decision trees organize attributes by arranging them by their values. RF executes feature selection, each tree calculates the significance of a feature based on its ability to improve the integrity of the leaves [71]. LIGHTGBM is a gradient boosting algorithm that uses decision trees. Different data points play different roles in the computation of the information gain. Higher gradient data points will have a greater influence on the information acquisition [49]. ET algorithm generates a large number of the unpruned decision or regression trees from the training dataset and then combines the predictions of multiple decision trees. ET possess similar properties as RF, randomly samples the features at each split point. Nevertheless, differs from RF, where it splits nodes by selecting cut-points entirely at random and fits each decision tree to the entire training dataset [50]. DT is a decision support technique that employs a binary model to display the hidden association between the input and outputs, also known as a pattern recognition capable of categorizing input variables into categories. It has some limitations such as failing to recognize the impact of weak attributes [51]. The KNN technique works on the principle that comparable/similar samples exist in the vicinity and have a higher probability. KNN approach finds distances between a new data point and all existing training data points, then selects the nearest data points (k) to the query [52]. LASSO is used for the regularization of data models. It conducts feature selection and can minimize model complexity while preventing overfitting. It executes L1 penalty, providing a type of automatic feature selection that has the effect of shrinkage of coefficients and thus input variables that do not provide much to the prediction set to zero [53]. EN is a standardized or penalized linear regression approach that is commonly used to solve high-dimensional feature space regression problems. EN automatically chooses variables, promoting continuous shrinkage [54].

Model validation: 10-fold cross-validation was performed on the training set (70%) to optimize the hyperparameters and to ensure robustness. The mean absolute error (MAE), root mean square error (RMSE) and coefficient of determination (R^2^) were employed to assess the performance of the models. In addition, the external data (30% of data) was used to assess the predictability of the models.

Attributes importance: A supervised approach that ranks features that are important for predicting the outcome was performed once the best model was selected [55]. The value of attributes varies from 0 to 1, with 1 representing the most information gain. In a regression model, the concept of “variance reduction” is used to guide the feature selection for internal nodes. Thus, we can evaluate how each feature reduces the impurity of the split (the feature with the highest reduction is chosen for the internal node). We measure the average impurity reduction for each feature across all trees in the RF model representing the feature relevance:

$$I_{g\;}\left(f\right)=\underset{M}{\sum }w_{p,n\;}\underset{M}{\sum }\Delta i_{f\;\left(\tau \;,\;M\right)}$$

where $I_{g}$ is the Gini importance which identifies the frequency of a particular feature $\left(f\right)$ chosen for the split and the importance of the feature’s value. Gini impurity reduction resulting from any peak split $\Delta i_{f\;\left(\tau \;,\;M\right)}$ is assembled individually for each feature nodes τ in the M number of weighted trees in the forest [71].

## 5. Conclusions

Researchers from multiple domains are interested in the application of nano-antioxidants for the prevention and treatment of oxidative stress-mediated disorders. We demonstrate the application of an RF algorithm to predict the efficiency of nano-antioxidants. The attribute importance analysis demonstrated the NMs’ P-chem characteristics such as type and core-size to have a significant impact on the predictions along with the exposure concentration (dosage). Furthermore, this paper emphasizes the significance of a consistent and systematic reporting structure and comparable measurement techniques indicating that their absence can result in an obstacle for modelling purposes. This is an important finding for policymakers and research funding bodies alike. The ability to predict functionalities represents a great opportunity in the healthcare field. Additionally, it will assist insurers and other stakeholders in the healthcare ecosystem to determine whether it is safe and beneficial (profitable) to invest in the development of nano-antioxidant therapies. Future research should therefore attempt to address this challenge through data capturing templates from empirical researchers that facilitate the construction of a comprehensive dataset.

## Figures and Tables

**Table 1:** The primary and final input variables in dataset I and dataset II.

			DATASET I		DATASET II		
Category	Variables	Type	Min-Max or Labels	Data Transformation		Methods of Determination	Missing Values %
Physico-chem properties	Core Size	Numeric	3.7–313 (nm), NaN	Selected	3.7–313 (nm), NaN	SEM, EDX, TEM, DLS, etc.,	3.06
	Hydrodynamic Size		32–752 (nm), NaN	Eliminated due to high NaN	_	DLS	78.30
	Encapsulation Efficiency		59.7–237 (%), NaN		_	DLS	90.34
	Zeta Potential		−20.6–46.8 (mV), NaN		_	DLS	65.09
	Polydispersity Index		0.145–1.76, NaN		_	DLS	82.05
	Surface Area		52.2–135.5 (m2/g), NaN		_	_	99.51
	Dosage		0– 200,000 (µg/mL), NaN	Selected	0–200,000 (µg/mL), NaN	_	9.86
	Synthesis Technique	Nominal	Green, Chemical	Selected	Green, Chemical		_
	Shape		Spherical, Hexagonal, Rod, Spherical/Rod, Dendrimer, Triangular, Cubic	Selected	Spherical, Hexagonal, Rod, Spherical/Rod, Dendrimer, Triangular, Cubic	TEM, HRTEM, SEM, FESEM, EDX, XRD	15.68
	NMs’ Type		Ag, ZnTe, CdTe, Chs, Se, Nd/ZnO, Au, Cu, CdSe, Chs/AL, AL, NaCas/GA, TiO_2_, MgO, etc.,	Selected	Ag, ZnTe, CdTe, Chs, Se, Nd/ZnO, Au, Cu, CdSe, Chs/AL, etc.,	XRD, EDX, SEM, FESEM, TEM, HRTEM, etc.,	_
	Coating		Allium sativum, areca catechu nut, Carboxymethyl cellulose (CMC), carboxymethyl cellulose (CMC)/curcumin, CCS, etc.,	Simplified: Transformed into Binary	Coated, Uncoated	SEM, EDX, TEM, DLS, XRD, HRTEM, STEM, FTIR	_
Exposure	Medium Used for Antioxidant Evaluation	Nominal	Extract, Solution, Emulsion, Food, Nanocomposites, Coating films	Selected	Extract, Solution, Emulsion, Food, Nanocomposites, Coating films	_	_
	Temperature	Numeric	0–37 (°C), NaN	Eliminated due to high NaN	_	_	76.33
	pH		7–7.4, NaN		_		98.62
	Thermal Stability		12–600, NA		_	TGA	86.59
	Spectrophotometer Absorbance		517–540, NaN	Selected	517–540, NaN	_	_
	Duration		0–2190 (h), NaN	Selected	0–2190 (h), NaN	_	_

## Data Availability

Dataset available at: 10.5281/zenodo.6584826, accessed on 26 May 2022.

## Supplementary Materials

The following supporting information can be downloaded at: https://www.mdpi.com/article/10.3390/ijms24032792/s1.
