# Supplementary material for: Employing Supervised Algorithms for the Prediction of Nanomaterial’s Antioxidant Efficiency
Source: Int J Mol Sci. 2023 Feb 1;24(3):2792. doi: 10.3390/ijms24032792 (PMC9918003; doi:10.3390/ijms24032792)
Supplement: Supplementary file 1 [file ijms-24-02792-s001.zip › ijms-2132994-supplementary.pdf]

Employing supervised algorithms for prediction of antioxidant capacity of nanoforms.

Mahsa Mirzaei[1], Irini Furxhi[*1,2], Finbarr Murphy[1], Martin Mullins[1]

1 Dept. of Accounting and Finance, Kemmy Business School, University of Limerick, Ireland. V94PH93
2Transgero Limited, Newcastle West, Limerick, Ireland
**Correspondence:*
mahsa.mirzaei@ul.ie. Tel: +353 87 3412425
irini.furxhi@ul.ie. Tel: +353 85 106 9771
finbarr.murphy@ul.ie. Tel: +353 86 108 8137
martin.mullins@ul.ie. Tel: +353 85 108 6426

Table S1. *Assessing the accuracy and performance of the models' predictions with inclusion of zeta potential variable. Comparison of seven regression models, using the test (30% data holdout). Columns used for this evaluation include type of NMs, medium use, core-size, shape, Zeta potential, synthesis techniques, coating, dosage, absorbance, duration and DPPH).*

Model	MAE	RMSE	R2
RF	8.9	14.6	0.75
ET	9.2	14.8	0.73
LIGHTGBM	10.5	15.3	0.72
DT	9.6	15.5	0.71
KNN	12.8	18.8	0.58
LASSO	17.9	21.7	0.44
EN	21.8	25.2	0.25

Table S2. *Assessing the accuracy and performance of the models' predictions with inclusion of zeta potential and after dropping shape variables. Comparison of seven regression models, using the test (30% data holdout). Columns used for this evaluation include type of NMs, medium use, core-size, Zeta potential, synthesis techniques, coating, dosage, absorbance, duration and DPPH).*

Model	MAE	RMSE	R^2
RF	8.9	14.6	0.78
ET	9.5	15.2	0.76
LIGHTGBM	11.2	16.5	0.72
DT	10.3	17.2	0.70
KNN	13.5	20.1	0.58
LASSO	18.4	22.6	0.48
EN	22.9	26.5	0.29

Table S3. *Assessing the accuracy and performance of the models' predictions after dropping shape. Comparison of seven regression models, using the test (30% data holdout). Columns used for this evaluation include type of NMs, medium use, core-size, synthesis techniques, coating, dosage, absorbance, duration and DPPH.*

Model	MAE	RMSE	R^2
RF	7.9	13.6	0.79
ET	8.6	13.9	0.78
LIGHTGBM	10.3	14.6	0.77
DT	9.2	15.7	0.73
KNN	12.1	18	0.65
LASSO	17.9	21.5	0.5
EN	21.9	25.2	0.32
